# Editorial: Current Devices in Mitral Valve Replacement and Their Potential Complications

**DOI:** 10.3389/fcvm.2022.891914

**Published:** 2022-04-26

**Authors:** Nicola Buzzatti, Maurizio Taramasso, Rishi Puri, Jan Van Der Heyden

**Affiliations:** ^1^Department of Cardiac Surgery, San Raffaele Scientific Institute, Milan, Italy; ^2^Department of Cardiac Surgery, HerzZentrum Hirslanden Zurich, Zurich, Switzerland; ^3^Cliveland Clinic, Cleveland, OH, United States; ^4^AZ Sint-Jan Brugge – Oostende AV, Brugge, Belgium

**Keywords:** mitral, replacement, transcatheter, repair, regurgitation

When the first ever percutaneous mitral valve replacement was performed on a patient in 2012 it raised great enthusiasm as well as the expectations to follow swiftly the road already laid down by transcatheter aortic valve implantation, expanding rapidly to a wide patient population, and growing year after year to become soon a routine procedure. Several different devices have been developed over the past decade to fulfill these promises.

In reality, compared to the aortic valve, the mitral is indeed a much more complicated apparatus with specific clinical, anatomical, and technical challenges which soon became very clear, such as the big dimensions of the mitral valve, the difficult anchoring, the anatomical interference the surrounding structures and the functional interdependence with the left ventricle. Such challenges proved difficult to overcome and indeed, after 10 years, only a minority of highly selected patients have been treated with transcatheter mitral replacement worldwide.

The purpose of the present anthology is to present the devices currently available for transcatheter mitral replacement along with their positive outcomes as well as their potential complication and discuss the challenges the field is facing to progress further in the next future.

Gheorghe et al. present a complete review of the most important devices developed, detailing the technical aspects which differentiate each one from the other, reporting their main outcomes as well as their possible complications. Some complications, such as the risk of left ventricle outflow tract obstruction are shared by all devices, while some others can be peculiar to each different replacement technology.

The transapical Tendyne valve is currently the only CE approved device for mitral replacement and indeed the one with the biggest experience in the clinical setting. Importantly this therapy can be applied not only to pure mitral regurgitation but also to selected cases of mixed regurgitation/stenosis as well as patients with severe mitral annular calcification, who represent one of the most challenging setting for both surgery and transcatheter devices. A focus on the Tendyne valve is provided by Dahle.

The blood flow around the mitral valve, slower than in the aorta, and the bulky design and fabrics of the transcatheter replacement devices make thrombosis a typical complication of such procedures during follow-up. Ascione and Denti discuss with available data the reasons why transcather mitral prostheses carry a heavy thrombotic burden as well as recommendations to prevent and to treat protheses thrombosis.

Kargoli et al. provide further details on the different complications after transcatheter mitral valve replacement. The management of each complication is described. Advanced interventional techniques may be required in some peculiar extreme cases but a patient-tailored multidisciplinary Heart-Team approach is always the common base to deal with such complex situations and help prevent issues in the first place.

Surgical mitral repair, when appropriate, is always to be preferred to surgical replacement. While the role of transcatheter mitral replacement in comparison to transcatheter mitral repair remains to be determined, the current excellent results of transcatheter mitral repair, especially in terms of safety, further raise the quality bar that replacement needs to meet to be considered a valid alternative. Indeed percutaneous mitral repair, rather than surgery, is currently the first competitor of transcatheter mitral replacement. Russo et al. discuss the repair vs. replacement dilemma while also elaborating on future developments and directions of replacement therapies.

Transcatheter mitral replacement is a precious option for patients who are not eligible for transcatheter mitral repair nor conventional surgery. Unfortunately its adoption is currently still bound to specific anatomies and technological limitations. Such challenges will require time to be fully overcome and indeed we are just still facing the early phase of the field but significant new mitral knowledge has already been acquired over the past decade thanks to transcatheter mitral replacement that will be the ground for future further improvements and developments. The road ahead is still long and it is not straightforward but the goal remains to allow more patients to be able to benefit from a safe and effective percutaneous mitral treatment, and it is well worth it.

## Author Contributions

All authors listed have made a substantial, direct, and intellectual contribution to the work and approved it for publication.

## Conflict of Interest

JV was employed by AZ Sint-Jan Brugge – Oostende AV, Brugge, Belgium. The remaining authors declare that the research was conducted in the absence of any commercial or financial relationships that could be construed as a potential conflict of interest.

## Publisher's Note

All claims expressed in this article are solely those of the authors and do not necessarily represent those of their affiliated organizations, or those of the publisher, the editors and the reviewers. Any product that may be evaluated in this article, or claim that may be made by its manufacturer, is not guaranteed or endorsed by the publisher.

